# A Case Report of Nitrous Oxide-induced Myelopathy: An Unusual Cause of Weakness in an Emergency Department

**DOI:** 10.5811/cpcem.1549

**Published:** 2023-07-19

**Authors:** Madeleine S. Birch, Emmelyn J. Samones, Tammy Phan, Mindi Guptill

**Affiliations:** Loma Linda University Medical Center, Department of Emergency Medicine, Loma Linda, California

**Keywords:** Vitamin B_12_ neuropathy, nitrous oxide, weakness, case report

## Abstract

**Introduction:**

Weakness is a common symptom that within itself does not indicate a specific diagnosis. Recreational inhalant use such as nitrous oxide (NO) may not often be disclosed. Additionally, professional or occupational history, such as being a dentist or dental assistant, should be determined because of higher reported rates of NO misuse.^1^ Nitrous oxide can cause vitamin B_12_ deficiency and resulting neuropathy. Nitrous oxide toxicity can have a wide variation of presentations with or without laboratory abnormalities or remarkable imaging findings, which can further complicate a diagnosis of weakness secondary to NO use.

**Case Report:**

A 33-year-old female presented to the emergency department with progressive bilateral leg numbness and objective weakness after repeated recreational NO use. After an extensive workup, she was found to have vitamin B_12_ deficiency and an electromyography study consistent with myeloneuropathy, despite normal imaging. She was prescribed high-dose vitamin B_12_ therapy and stopped using NO. One year after diagnosis, our patient maintained NO sobriety and had near-complete resolution of prior neurologic deficits.

**Conclusion:**

The use of recreational inhalant and the patient’s occupation should be considered when a patient presents with weakness. Obtaining vitamin B_12_ and methylmalonic acid levels should be considered for diagnosis. However, NO-induced neuropathy can be seen in patients with normal vitamin B_12_ and methylmalonic levels and patients do not always have abnormal imaging findings. The healthcare team should consider the varied presentations and findings of substance-induced conditions such as NO toxicity.

## INTRODUCTION

Weakness is a common presenting chief complaint of patients seeking care in the emergency department (ED), making up an estimated 10% of annual visits.^2^ One under-recognized cause of weakness is recreational use of inhalants. Nitrous oxide (NO) use is increasing, particularly among music festival and club attendees with a prevalence of 38% in 2014.^3^ While weakness is a vague complaint with a broad differential, careful history-taking and consideration of substance use and occupational history can assist in the diagnosis and treatment of underlying neurological pathology. Neurotoxicity from NO is a potentially reversible condition that can be treated with appropriate vitamin B_12_ supplementation and abstinence from NO use.

## CASE REPORT

A 33-year-old woman employed as a warehouse associate without significant past medical history presented to the ED with bilateral lower extremity weakness that had been progressive for one month. The patient reported that symptoms initially began as a “pins and needles” sensation in her feet that slowly progressed to a “heavy” sensation and difficulty walking. She denied recent trauma, illness, or pain and disclosed she regularly huffed 20–30 “balloons” of NO two to three times a month. On physical exam, she had normal vital signs, cranial nerve function, and rectal tone. She had decreased bilateral lower extremity strength with decreased sensation to pinprick, vibration, and pain to the mid-abdomen with full sensation and strength preserved in her upper extremities. In addition, she had a positive Lhermitte’s sign and Romberg test with diminished, 1+ patellar and Achilles reflexes with 2+ brachioradialis, and biceps and triceps reflexes bilaterally. The patient was admitted to the neurology ward.

Laboratory results obtained after admissions were notable for a decreased vitamin B_12_ level with an elevated methylmalonic acid level without macrocytosis, normal levels of intrinsic factor without antibodies, and unremarkable cerebrospinal fluid studies with normal inflammatory and infectious markers. Folate and copper levels were also found to be normal. Radiographic findings including contrasted magnetic resonance imaging brain and spine were normal. Electromyography studies did not suggest demyelination; however, they were abnormal and suggestive of myeloneuropathy in the setting of vitamin B_12_ deficiency from NO use.

The patient was administered high-dose vitamin B_12_ injections (1,000 micrograms daily) with significant clinical improvement. The patient was discharged home on day three of hospitalization with a walker and outpatient physical therapy. At eight-week neurology follow-up, the patient was able to ambulate independently with 5/5 strength in bilateral hip flexors with negative Romberg. The patient had full resolution of symptoms one year after initial onset. She maintained sobriety from NO, and her vitamin B_12_ and methylmalonic acid levels have remained normal.

## DISCUSSION

Vitamin B_12_ is a fat-soluble enzyme found in animal products. Once absorbed, vitamin B_12_ becomes an essential cofactor for enzymes involved in deoxyribonucleic acid, fatty acids, and myelin synthesis; thus, B_12_ deficiencies can present with hematological or neurological deficits. Vitamin B_12_ is an important cofactor in the conversion of methylmalonyl-CoA to succinyl-CoA (Figure). In patients with vitamin B_12_ deficiencies, levels of methylmalonic acid and homocysteine levels accumulate and are thought to damage myelin, resulting in neurologic deficit.^4^ Nitrous oxide, also known as “laughing gas,” is an odorless gas that is used recreationally for euphoric effect. Nitrous oxide causes permanent oxidation of vitamin B_12_, rendering it useless as a cofactor for essential enzymatic processes resulting in deficiency, which can present as a myeloneuropathy or subacute combined degeneration of the spinal cord.^5^ In a systematic review conducted by Garakani et al., 72 of 91 patients presented with neurological sequelae, with radiographic findings in 39 of those 72 patients. Vitamin B_12_ deficiency was reported in 52 of the 72 patients, and elevated methylmalonic acid and homocysteine levels were reported in several patients.^6^

**CPC-EM Capsule**

What do we already know about this clinical entity?

*Nitrous oxide can cause vitamin B* *_12_* *deficiency. vitamin B* *_12_* *is an essential enzyme for neuronal health and deficiencies can present with neurological deficits.*

What makes this presentation of disease reportable?

*The patient presented had vitamin B* *_12_* *deficiency and an electromyography study consistent with myeloneuropathy, despite normal imaging.*

What is the major learning point?

*Neurotoxicity from nitrous oxide use can vary in clinical presentations, laboratory findings and imaging findings. High dose Vitamin B therapy is the treatment.*

How might this improve emergency medicine practice?

*Recreational inhalant use and occupational exposure should be considered in patients presenting with weakness regardless of normal imaging.*

Recent studies report recreational NO use has been increasing, and NO toxicity can have varied presentations and recovery of neurological symptoms.^4^,^8^ When symptomatic, vitamin B_12_ levels can be low as in this case; however, neurological symptoms can still occur in patients with normal vitamin B_12_ levels.^6^ Neurological manifestations can include ataxia, paresthesias, polyneuropathy, subacute combined degeneration, or myelopathy. Patients can also present with psychiatric manifestations such as hallucinations and delirium.^9^

## CONCLUSION

Neurotoxicity from nitrous oxide use can present with a wide variety of clinical presentations, laboratory findings, and imaging findings. While neurological deficits can vary in severity and in recovery, supplemental vitamin B_12_ and complete cessation of NO use is the recommended treatment. As previously noted, symptomatic weakness does not lend itself to a specific diagnosis and requires the clinician to maintain a broad differential including atypical causes such as nutritional deficiencies, nutritional toxicities, occupational exposure, or inhalational drug use.

## Figures and Tables

**Figure:**
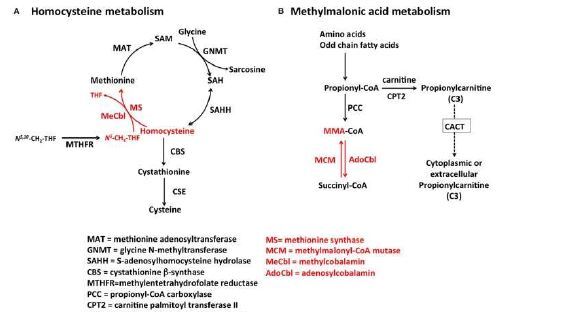
Methylmalonic acid and homocysteine metabolism,^7^ from Hannibal et al. Biomarkers and algorithms for the diagnosis of Vitamin B12 deficiency. Reproduced with author permission.
